#  Developmental expression of a functional TASK-1 2P domain K^+ ^channel in embryonic chick heart

**DOI:** 10.1186/1423-0127-16-104

**Published:** 2009-11-23

**Authors:** Hengtao Zhang, Jeremy Parker, Neal Shepherd, Tony L Creazzo

**Affiliations:** 1 The George and Jean Brumley, Jr, Neonatal-Perinatal Research Institute, Division of Neonatology/Department of Pediatrics, Duke University Medical Center, Box 2635, Durham, NC 27710, USA

## Abstract

**Background:**

Background K^+ ^channels are the principal determinants of the resting membrane potential (RMP) in cardiac myocytes and thus, influence the magnitude and time course of the action potential (AP).

**Methods:**

RT-PCR and *in situ *hybridization are used to study the distribution of TASK-1 and whole-cell patch clamp technique is employed to determine the functional expression of TASK-1 in embryonic chick heart.

**Results:**

Chicken TASK-1 was expressed in the early tubular heart, then substantially decreased in the ventricles by embryonic day 5 (ED5), but remained relatively high in ED5 and ED11 atria. Unlike TASK-1, TASK-3 was uniformly expressed in heart at all developmental stages. *In situ *hybridization studies further revealed that TASK-1 was expressed throughout myocardium at Hamilton-Hamburger stages 11 and 18 (S11 & S18) heart. In ED11 heart, TASK-1 expression was more restricted to atria. Consistent with TASK-1 expression data, patch clamp studies indicated that there was little TASK-1 current, as measured by the difference currents between pH 8.4 and pH 7.4, in ED5 and ED11 ventricular myocytes. However, TASK-1 current was present in the early embryonic heart and ED11 atrial myocytes. TASK-1 currents were also identified as 3 μM anandamide-sensitive currents. 3 μM anandamide reduced TASK-1 currents by about 58% in ED11 atrial myocytes. Zn^2+ ^(100 μM) which selectively inhibits TASK-3 channel at this concentration had no effect on TASK currents. In ED11 ventricle where TASK-1 expression was down-regulated, I_K1 _was about 5 times greater than in ED11 atrial myocytes.

**Conclusion:**

Functional TASK-1 channels are differentially expressed in the developing chick heart and TASK-1 channels contribute to background K^+ ^conductance in the early tubular embryonic heart and in atria. TASK-1 channels act as a contributor to background K^+ ^current to modulate the cardiac excitability in the embryonic heart that expresses little I_K1_.

## Background

TASK-1 is an acid-sensitive two pore domain potassium channel (K2P) that is activated by alkaline pH and is inhibited by external protons [1]. Activation of TASK-1 channels produces an outwardly rectifying current at normal physiological condition. The currents do not show time- and voltage-dependent activation, inactivation or deactivation [2,3]. With a pK_a _of 7.3, TASK-1 channels could be open throughout an AP of cardiac cells and therefore contribute to repolarization of APs as well as to the RMP. In addition to its pH sensitivity, TASK-1 channel is also sensitive to oxygen and local anesthetics and is tightly regulated through protein kinases A, C, G and phospholipase C signaling pathways [4,5]. Thus, the functional role of TASK-1 channel will depend to a great extent on where it is expressed.

TASK-1 channel is highly expressed in heart [1,6-10]. By means of real time PCR, Liu et al shows that TASK-1 is one of the predominant K2P channels and is expressed more in the atria than in the ventricles of both embryonic and adult rat heart [10]. In adult mouse heart, TASK-1 is expressed in the atria but not in the ventricles [1]. We have characterized the cardiac expression of TASK-1 channel in developing chick and mouse hearts with immunofluorescent staining [6]. The expression pattern of TASK-1 channel in chick embryonic heart was similar to that in the developing mouse heart, where TASK-1 channels were expressed ubiquitously in the tubular heart but later in development it was restricted to ventricular conduction system.

We were intrigued by the expression of TASK-1 channels throughout the heart tube in early developmental stages. In the early tubular heart, all myocytes including ventricular myocytes exhibit spontaneous electrical activities similar to adult sinoatrial nodal cells. The lack of inward rectification K^+ ^currents (I_K1 _or Kir channels) likely contributes to their automaticity [11]. However, a small background K^+ ^conductance is necessary otherwise the myocytes would be too depolarized to beat spontaneously. Our hypothesis is that TASK-1 channels could be one of the contributors to this small background K^+ ^conductance. Although the functional role of TASK-1 channel in developing heart is still unknown it seems likely to be involved in setting RMP and possibly influencing the shape of action potentials [8].

In this report, we further studied the distribution of TASK-1 and its electrophysiology during chicken heart development. Consistent with our previous studies, TASK-1 was highly expressed in the tubular heart early in development. In later development, expression of TASK-1 was retained in the atria but was down-regulated in the ventricle. Likewise, patch clamp studies showed that acid sensitive background K^+ ^currents were present in S14 tubular heart and in ED11 atria, but little in ED5 and ED11 ventricle. Conversely, there was little or no I_K1 _in ED11 atrial myocytes while I_K1 _was substantial in ED11 ventricular myocytes. These results demonstrate that TASK-1 channels contribute to background membrane K^+ ^conductance where inwardly rectifying K^+ ^currents (I_K1_) are not present in the developing heart.

## Methods

### Total RNA isolation and RT-PCR of TASK-1 and TASK-3

Whole S11 chick embryos (40 hour incubation at 37°C), heart tubes from S14 (50 hours) and S18 (3 day incubation), ED5 and ED11 hearts were dissected and collected in DEPC-treated PBS solution. The ventricles and atria of ED5 and ED11 embryos were separated under a microscope. The dissected tissues were immediately homogenized in the presence of β-mercaptoethanol (β-ME, Sigma). The total RNAs were prepared by using the Qiagen RNeasy kit (Qiagen). Genomic DNA was removed by on-column treatment of RNase-free DNase I (Roche Inc.). 1 μg DNase I treated total RNA was used to synthesize the first strand cDNA with Invitrogen Platnium III^® ^reverse transcriptase (Invitrogen Inc). The reverse transcription was terminated by heating the reactions to 85°C for 15 min and then diluted 10 times prior to PCR reactions.

The specific primers for chicken TASK-1 were designed from the cloned cDNA sequence (accession number DQ272256, sense primer 5'-CTCTTTCTACTTCGCCATCAC-3' and antisense primer 5'-CTTCTCGTCCTCGGCGTTCA-3'). Primers for chicken TASK-3 were designed from the predicted cDNA sequences (accession number XM_417369, sense 5'-TCTACTTCGCCATCACCGTCAT-3' and antisence 5'-CTCGTTCTTCTGCAGGGCCAC-3'). Glyceraldehyde-3-phosphate dehydrogenase (GAPDH) was used as an internal control (sense 5'-TGGGAAGCTTACTGGAATGG-3' and antisense 5'-ACCAGGAAACAAGCTTGACG-3', accession number: NM_204305). For PCR reaction, a total of 25-35 cycles for melting, annealing and extension steps (95°C, 25 s; 55-60°C, 30 s and 72°C, 45 s) were carried out. 10 μL amplification products were checked on 1.5% agarose gel. A no template (NTC) was used as a negative control to monitor the primer dimerization and non-specific amplification. PCR products were further cloned into pGEM T-easy vector for sequencing and synthesizing cRNA probe for *in situ *hybridization studies.

#### 2.2. Digoxin-labeled cRNA probe synthesis and in situ hybridization

The plasmid DNA containing chicken TASK-1 clones was linearized and then used to synthesize Dig-labeled cRNA probe by using either SP6 or T7 RNA polymerase and following the manufacturer's instructions (Roche Biochemical Corp.).

For *in situ *hybridization, chick embryos of S11, S18 were collected in DEPC-treated PBS solution. For ED11 hearts, hearts were first excised and perfused through the aorta subsequently with DEPC-treated PBS and 4% paraformaldehyde under a dissection microscope. The collected embryos and hearts were immediately placed in pre-cooled 4% paraformaldehyde on ice and further fixed overnight on an orbital rotator at 4°C. The fixed embryos and embryonic hearts were then dehydrated in 50%, 75%, 100% (PBS:Methanol) and stored at -20°C. Prior to *in situ *hybridization, stored embryos and hearts must be re-hydrated. The hybridization was carried out at 70°C overnight in standard hybridization mix containing 1 μg/ml cRNA probe. After NBT/BCIP reactions, the stained embryos and hearts were processed for embedding in paraffin and sectioning with a thickness of 14 μm.

### Chick embryonic myocyte isolation

Chick embryonic myocytes were isolated by using enzymatic dissociation methods. Briefly, the atria and ventricles of ED5 and ED11 hearts were separated before the enzymatic digestion. The atria and ventricles of ED5 and ED11 hearts were then incubated for 10 minutes in Mg^2+ ^and Ca^2+ ^free Tyrode solution containing 0.1 mg/ml Trypsin, 1 mg/ml collagenase and 0.2 mg/ml bovine serum albumin at room temperature. Then, three digestion steps were subsequently carried out at 37°C. The enzyme digestion times were empirically adjusted to achieve maximum viability for each of the developmental stages. The dissociated myocytes were collected by centrifuging and re-suspended in Modified M-199 medium (Gibco Corp.). For the preparation of S14 myocytes, whole S14 tubular hearts were used for dissociation. The myocytes were incubated overnight in a plastic petri dish in which the myocytes did not attach. The myocytes were used within 24 hours after dissociation.

### Electrophysiological recordings

Embryonic myocytes were transferred to a Warner perfusion chamber (Warner Instruments) and were continuously perfused with Tyrode solution (S-1) at a flow rate of ~2 ml/min. For whole-cell patch clamp recordings of TASK currents, dissociated myocytes were initially bathed with Tyrode solution (S-1, Table 1). After the formation of whole cell configuration, bath solution was then switched to experimental solution either S-2 or S-3 (Table 1). Pipettes were pulled from 1.5 mm borosilicate glass using a P-97 Glass Microelectrode Puller (Sutter Corp.). Patch pipette usually had a resistance of 4-8 MΩ when filled with pipette solution P-1 or P-2 (Table 1). The liquid junction potential was cancelled before pipette contacted the cells. When most of external chloride was replaced by aspartate, an agar bridge (3 M KCl) was used to reduce junction potential. The membrane and access resistances were monitored during experiments by means of membrane test in Axon Clampex 8.0 software. Only those cells that had a seal resistance larger than 1 GΩ were used for the final analysis. Whole-cell currents were sampled at 5 kHz and recorded with an AXOPATCH 200B voltage-clamp amplifier (Axon instrument). The command voltage steps were applied with pCLAMP 8.0 and a DIGIDATA 1322A interface (Axon Instrument). Data were stored on the hard disk for off-line analysis. All experiments were carried out at room temperature except where specified.

**Table 1:** The components of solutions used in patch-clamp experiments

Components (in mM)	S-1	S-2	S-3	P-1	P-2
NaCl	140	-	-	-	
KCl	5.4	125	-	145	
CaCl_2_	1.8	-	-	-	5
MgCl_2_	1.05	2	2	2	1
Dextrose	5.5	5.5	5.5	-	-
HEPES	10	10	10	10	10
EGTA	-	-	-	0.5	11
K-Aspartate	-	-	140	-	130
ATP·Mg	-	-	-	5	-
ATP·Na_2_	-	-	-	-	5
BaCl_2_	-	0.1	0.1	-	-
TEA·Cl	-	20	10	-	-
4-AP	-	0.5	0.5	-	-

Note: pH of pipette solutions (P-1 and P-2) was adjusted to 7.2 with KOH. For all bath solutions pH was adjusted to 7.4 with NaOH or KOH. TEA: tetraethylammonium, 4-AP: 4-aminopyridine.

All chemicals were purchased from Sigma except where specified. Anandamide and ZD-7288 were purchased from Tocris Bioscience (Tocris. MO). A 3 mM stock solution of anandamide was prepared in 100% ethanol and stored in freezer. The working solution of anandamide was freshly prepared before each experiment. In all anandamide experiments, control solutions contained appropriate concentrations of ethanol.

### Data Analysis

All data are presented as mean ± S.E. For statistical analysis, a paired or unequal variance student T-test was used to determine statistical significance. A p value < 0.05 was considered to be significant and was indicated with asterisk and non-significance by NS.

## Results

### Developmental expression of TASK-1 in chick embryonic heart

**Figure 1:**
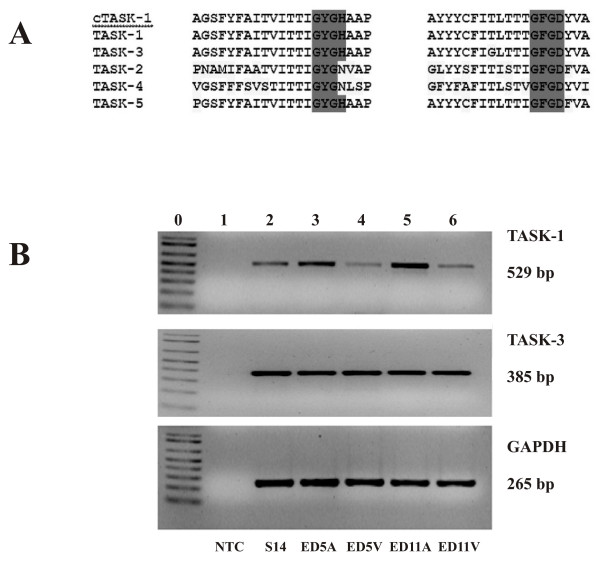
**Expression of TASK-1 during chick heart development by RT-PCR**. A: amino acid sequence alignment of pore regions of TASK-1 with all human TASK channels. B: TASK-1 and TASK-3 RT-PCR: Lane 0 shows 1 Kb DNA markers. Lane 1-6 as indicated at bottom (see text for definition). GAPDH expression was used as a reference and was equivalent in all samples after 25 to 40 cycle two step RT-PCR amplification. The size of cDNA products are indicated on the right hand and the developmental stages are labeled at the bottom.

A distinguishing characteristic of K2P channels is that the ion selectivity filter structure (GYGX) is not strictly conserved. A single mutation will change the ion selectivity of K2P channels [12-14]. We therefore compared chicken TASK-1 pore regions with those members of human TASK family as shown in Fig. 1A. The shaded region indicates the signature sequences of K^+ ^ion selectivity filter in both pore regions. Chicken TASK-1 has an identical pore amino acid sequences with human TASK-1, indicating conservation of TASK-1 gene. The developmental expression of TASK-1 and TASK-3 were then studied by RT-PCR. TASK-1 was expressed in early heart development S14, but was dramatically reduced in ED5 and ED11 ventricles (Fig. 1B). However, expression of TASK-1 in chick ED5 and ED11 atria remained relatively high. Unlike TASK-1, TASK-3 is equally expressed in the heart developmental stages studied in this report (Fig. 1B).

**Figure 2:**
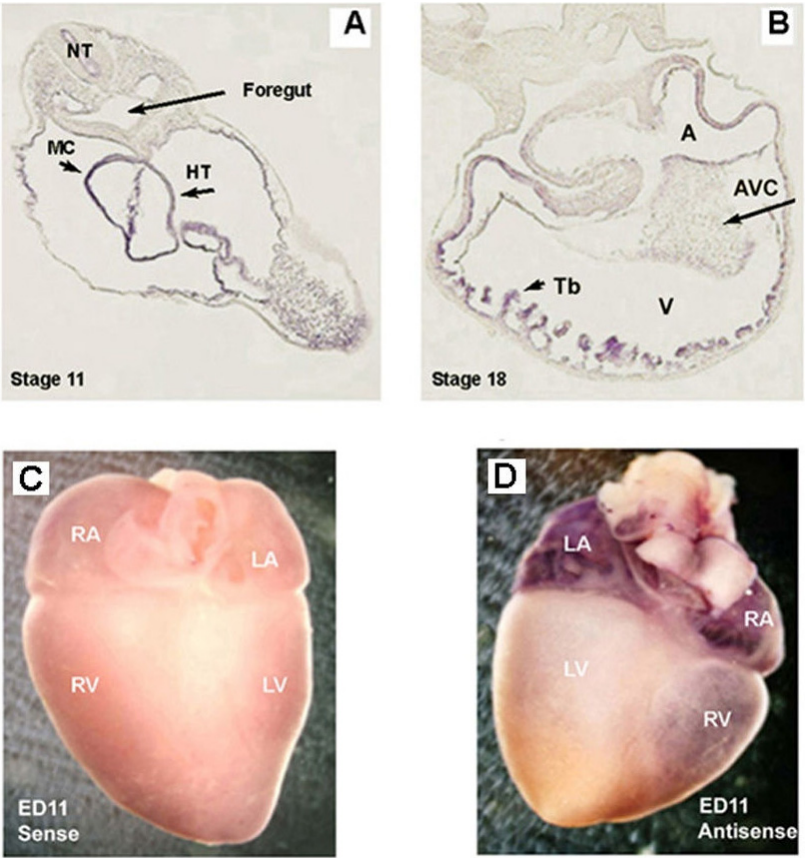
***In situ *hybridization of chicken TASK-1 expression in developing chick heart**. A: *in situ *hybridization of S11 chick embryos. TASK-1 was ubiquitously expressed in S11 embryonic heart. B: *In situ *hybridization of TASK-1 in S18 chick embryonic heart. TASK-1 was expressed in the both the atrium and ventricle. However, expression appeared greatest in the trabeculae of the S18 heart. Whole mount *In situ *hybridization of TASK-1 in ED11 chick heart shown in C and D. C: a control in situ hybridization with sense RNA probe staining. No detectable purple staining was observed. D: TASK-1 was strongly expressed in the both right and left atria and lightly expressed in the ventricle in ED11 chick heart. Abbreviations are: heart (HT), myocardium (MC), neural tube (NT), trabeculae (Tb), atrial-ventricular cushion (AVC), right atrium (RA), left atria (LA), right ventricle (RV) and left ventricle (LV).

Although we have used immunohistochemistry to characterize the cardiac expression of TASK-1 in chick embryonic heart there is still concern about the specificity of commercial available antibodies of TASK-1 channels [3]. For this reason, we determine the regional expression of TASK-1 during heart development by means of *in situ *hybridization (ISH). TASK-1 was ubiquitously expressed in the myocardium of the early heart at S11 (Fig 2A). In S18 chick heart, TASK-1 was most strongly expressed in trabeculae, where the ventricular conducting system develops (Fig. 2B). Further, TASK-1 was heavily expressed in ED11 atria and was slightly expressed in the right ventricle (Fig. 2D). No TASK-1 expression was visible in ED11 left ventricle. A typical control using sense RNA probe in chick ED11 embryonic heart was shown in Fig. 2C, indicating the high specificity of antisense TASK-1 probe.

### Developmental regulation of TASK-1 currents in chick embryonic myocytes

Based on the results of our expression studies, we measured TASK currents in chick embryonic myocytes and the contribution of two major acid-sensitive K2P channels, TASK-1 and TASK-3, by their differential pH sensitivity (pK_a _of 7.3 and 6.7, respectively). TASK currents inhibited by lowering pH from pH 8.4 to 7.4 are only contributed by TASK-1 channels while TASK-3 channels are still active in this pH range. However, in the pH range between 7.4 and 6.4, both TASK-1 and TASK-3 channels could contribute to TASK currents [15,16]. The TASK-1 or TASK-3 currents were also identified as those blocked by 3 μM anandamide or 100 μM zinc. At the concentrations used here anandamide and zinc selectively inhibit TASK-1 and TASK-3 currents, respectively [17,18].

**Figure 3:**
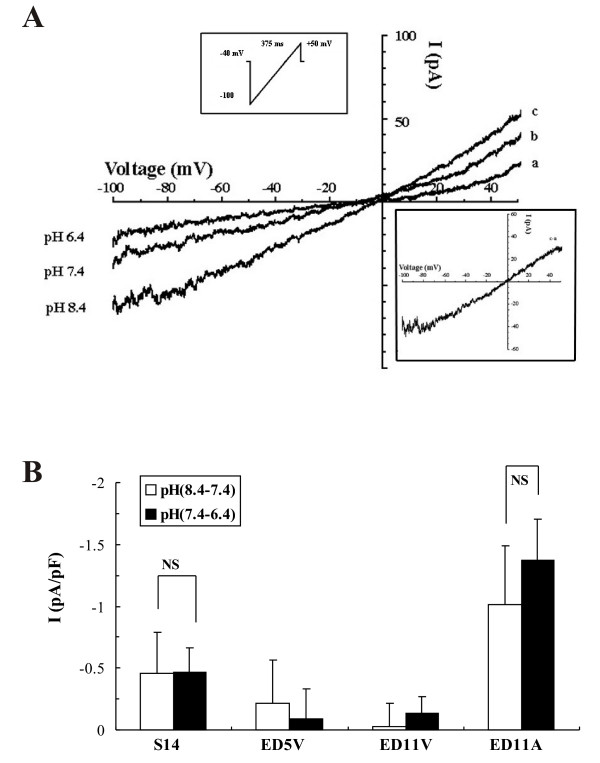
**Patch clamp studies of acid-sensitive K^+ ^currents (TASK currents) at different embryonic ages**. The background K^+ ^currents were activated with a 375 ms depolarizing ramp from -100 mV to +50 mV from a holding potential of -40 mV in an elevated [K^+^]_o _(125 mM). A: representative recordings of background K^+ ^currents in ED11 atrial myocytes and their pH sensitivity. The pH is indicated next to each trace. B: The difference currents between pH 8.4 and pH 7.4 (blank column) and between pH 7.4 and pH 6.4(filled column) at -80 mV at different developmental ages as indicated under each column. The data were averaged from *n *= 9 (S14), 3 (ED5 ventricle), 4 (ED11V), and 6 (ED11A) cells.

In the present study, TASK currents were recorded in external solutions with elevated [K^+^]_o _(125 mM). To avoid possible interfering K^+ ^currents, 100 μM Ba^2+ ^was used to inhibit inwardly rectifying K^+ ^currents. At this concentration, Ba^2+ ^has a minimal effect on TASK-1 currents [19]. 0.5 mM 4-AP was used to block transient K^+ ^current (I_to_). 10 or 20 mM tetraethylammonium (TEA) was used to inhibit the activation of delayed rectifier K^+ ^currents (I_K_). The hyperpolarization-activated pacemaker current I_f _was blocked by ZD-7288 (5 μM). Under these recording conditions, I-V curves of ED11 atrial myocytes displayed slight outward rectification (Fig. 3A) and showed pH sensitivity in elevated external [K^+^]_o _solution (S-2 and P-1). Consistent with PCR results, TASK-1 currents, measured by the current difference between pH 8.4 and 7.4 at -80 mV, decreased with development in embryonic ventricular myocytes but were maintained in embryonic atrial myocytes (Fig. 3B). Interestingly, TASK-1 currents (pH 8.4-7.4) and TASK currents (pH 7.4-pH 6.4) were not significantly different at any heart developmental stage, suggesting that TASK currents are mostly due to TASK-1 channels. However, ED11 atrial myocytes showed significantly higher TASK currents than ED11 ventricular myocytes (p < 0.01 for pH 7.4-6.4). Of all heart developmental stages used, ED11 atrial myocytes showed the greatest the acid-sensitive current densities (Fig. 3B). These results indicated that there are functional TASK-1 channels in embryonic heart.

**Figure 4:**
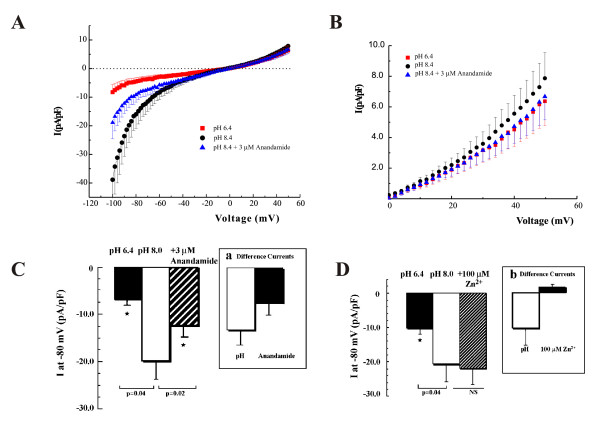
**Effect of anandamide, pH and Zn^2+ ^on TASK-like currents of ED11 atrial myocytes in Cl^- ^substituted solutions**. A: I-V curves averaged from 6 cells showing the effects of pH and anandamide on background K^+ ^currents in ED11 atrial myocytes during a 2s depolarizing ramp from -100 to +50 mV. The cells were held at -40 mV and perfused with 140 mM K-aspartate solution. Note TASK-like background K^+ ^current had a pronounced inward rectification at pH 8.0. B: The enlarged I-V curves at positive potential. C: The summarized data (*n *= 6 cells) of the effects of pH and anandamide on TASK-like background K^+ ^currents (at -80 mV). The background K^+ ^currents at pH 6.4 (filled column), pH 8.0 (blank column) and pH 8.0 in the presence of 3 μM anandamide (back slashed column) are as indicated. The inset panel (a) of Fig. 4C shows pH- (blank column) and anandamide- (filled column) sensitive background K^+ ^currents. The anandamide-sensitive currents account for 58% of TASK-like background K^+ ^currents. D: Zn^2+ ^had no effect on the TASK-like background K^+ ^currents. The summarized data (*n *= 11) of the effects of 100 μM Zn^2+ ^on TASK-like background K^+ ^currents in ED11 atrial myocytes as indicated. The inset panel b of Fig. 4D shows pH- (blank column) and Zn^2+^- (filled column) sensitive currents at -80 mV in ED11 atrial myocytes.

The contribution of TASK-1 channels to TASK currents was further studied in chick ED11 atrial myocytes simply because they had the highest current density. TASK currents were evoked by a 2 s depolarizing ramp from -100 mV to +50 mV (Fig. 4A). Both intracellular and external Cl^- ^were mostly substituted by aspartic acid (S-3 and P-2) to avoid the interference of acid sensitive Cl^-^ currents [20]. Under these experimental conditions, membrane currents showed inward rectification at negative potentials and slightly outward rectification at positive potentials. I-V curves are drawn from 6 cells with an average C_p _of 10.8 ± 1.3 pF (Fig. 4A). 3 μM anandamide abolished more than half of inward current at pH 8.0 at -80 mV (~58%, Fig. 4C). The pH sensitive currents (pH8.0 - pH6.4) at -80 mV were -13.1 ± 3.2 pA/pF and the mean of anandamide sensitive currents at -80 mV was -7.6 ± 2.6 pA/pF (The inset of Fig. 4C). Fig. 4B shows the I-V relationship of TASK currents at potentials between 0 and +50 mV (Fig. 4B). In this potential range, TASK currents activated by pH 8.0 were much smaller than those at negative potentials. However, TASK currents were inhibited by either lowering pH to 6.4 or 3 μM anandamide (Fig. 4B), indicating that in this potential range TASK currents were mostly due to TASK-1 channels. At +50 mV, the mean currents at pH 8.0, pH6.4 and in the presence of 3 μM anandamide were 7.87 ± 1.67, 6.37 ± 1.60 and 6.67 ± 1.52 pA/pF, respectively. This result is well consistent with pH sensitivity studies (Fig. 3B) showing that TASK currents between pH7.4 and pH6.4 are mostly due to the TASK-1 channel. Therefore, the currents activated by pH 8.0 and inhibited by pH 6.4 and anandamide are consistent with TASK-1 currents in chick embryonic heart.

Whether TASK-3 channels contribute to TASK currents was determined by using 100 μM Zn^2+^, at a concentration that selectively inhibits TASK-3 channels [17]. While inward currents activated by pH 8.0 at -80 mV were largely inhibited by pH 6.4 (blank column), 100 μM Zn^2+ ^did not inhibit TASK currents. In fact, it slightly increased TASK currents at -80 mV from -20.1 ± 5.7 to -21.5 ± 5.1 pA/pF (NS) (Fig. 4D). At -80 mV, the currents at pH 8.0 and pH 6.4 were -20.1 ± 5.7 and -9.6 ± 1.6* pA/pF, respectively (n = 9, C_p _= 8.6 ± 1.2 pF). The pH-sensitive inward currents were -10.5 ± 5.3 pA/pF and Zn^2+^-sensitive K^+ ^currents were 1.4 ± 1.1 pA/pF (Fig. 4D). Thus, 100 μM Zn^2+ ^has little effect on the inward currents, suggesting that TASK-3 channels contribute little or nothing to TASK currents. Since TASK-3 transcripts were found in chick embryonic heart, there remains the possibility that TASK-3 exists as a heterodimer with TASK-1 which may not be sensitive to 100 μM Zn^2+ ^but possibly to anandamide.

#### 3.3 I_k1 _is much larger in embryonic ventricular myocytes than in atrial myocytes and is inhibited by 100 μM Ba^2+^

**Figure 5:**
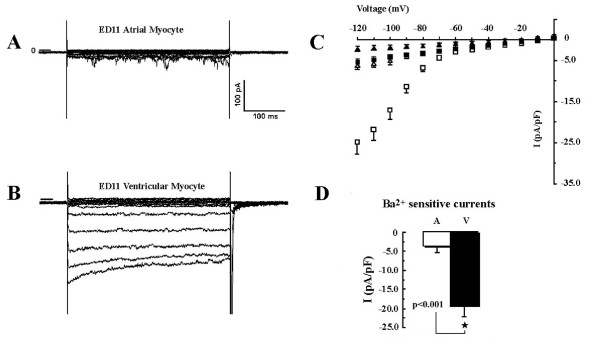
**Inwardly rectifying K^+ ^currents, I_K1_, in ED11 atrial and ventricular myocytes**. Panel A and B are representative I_K1 _current recordings from ED11 atrial and ventricular myocytes. The currents were evoked by depolarizing voltage steps between -120 and 0 mV with an interval of +10 mV from a holding potential of -40 mV in normal Tyrode solution. C: I-V relationships of I_K1 _in ED11 atrial and ventricular myocytes in the presence (black triangle and square) and absence (open triangle and square) of 100 μM Ba^2+^. The data are presented as Mean ± SE and are the average from 9 ED11 atrial myocytes, and 10 ventricular myocytes. D: Comparison of I_K1 _current densities of ED11 atrial and ventricular myocytes at -120 mV. The I_K1 _current densities were calculated as 100 μM Ba^2+ ^sensitive K^+ ^currents. At -120 mV, the I_K1 _current density in ED11 atrial myocytes is about 5 times less than in ventricular myocytes.

**Figure 6:**
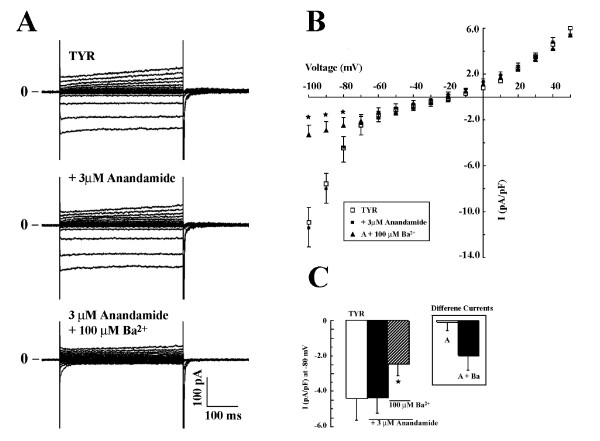
**Anandamide has no effects on inwardly rectifying K^+ ^current I_K1 _and delayed rectifier K^+ ^current I_K_**. A: typical current recordings from the same cell in Tyrode (on top left panel), in the presence of 3 μM Anandamide (middle), and in the presence of both anandamide and 100 μM Ba^2+ ^(bottom). The K^+ ^currents were evoked by applying 400 ms depolarizing steps from -120 to +50 mV with an interval of 10 mV from a holding potential of -40 mV. B. I-V relationships before (open square), after addition of 3 μM anandamide (black square), and Ba^2+ ^and anandamide together (black triangle). The asterisks indicate significance. The data were averaged from 5 cells. C: Summarized data (*n *= 11) of inwardly rectifying K^+ ^currents at -80 mV and the effects of anandamide and Ba^2+^. The horizontal bar indicates the drug administration. The blank column is the control in Tyrode, anandamide alone (black column), and Ba^2+ ^and anandamide together (column with back slash). Anandamide had no effect on K^+ ^currents in ED11 ventricular myocytes. The inset of Fig. 6C summarizes the anandamide and Ba^2+ ^sensitive currents. The data were presented as mean ± SE from the same cells (*n *= 11).

Our data suggest that TASK-1 currents are present in early embryonic heart and in ED11 atrial myocytes, but not in embryonic ventricles after ED5. In mature working myocardium, inwardly rectifying K^+ ^channels are dominant in setting the RMP. It is possible that the differential expression of TASK-1 is related to a differential expression of I_k1_. To examine this possibility, we compared the inwardly rectifying K^+ ^current, I_K1_, in chick ED11 atrial and ventricular myocytes. In these studies, normal Tyrode solution (S-1) was used and I_K1 _currents were identified as 100 μM Ba^2+^-sensitive currents (Figs. 5 and 6).

In ED11 atrial myocytes, there was very little inward current that could be easily identified as I_K1 _at normal physiological condition (Fig. 5A), while in ED11 ventricular myocytes (Fig. 5B) a large portion of inward current appeared to be due to I_K1_, having a steep inward rectification at negative potentials. This qualitative assessment was verified by the application of 100 μM Ba^2+^, which blocked a small amount of inward current in the atrium and a much larger amount in the ventricle (Fig. 5C). Ba^2+^-sensitive currents in ED11 atrial and ventricular myocytes at -120 mV are shown in Fig. 5D. At -120 mV, Ba^2+^-sensitive current densities in ventricular myocytes (-19.4 ± 2.7 pA/pF, *n *= 10) were significantly larger than in atrial myocytes (3.7 ± 1.5 pA/pF, *n- *= 4, p < 0.001). These data show that in ED11 heart, I_K1 _in the ventricles is about 4-5 times greater than in the atria.

Note that the apparent reversal potential of I-V curve (I_K1_, Fig. 5C) was about -20 mV, much more positive than is expected for the intra- and extra-cellular K^+ ^concentrations used in these experiments (S-1 and P-II, -80 mV). However, in 5 atrial cells replacement of Na^+ ^with NMDG shifted reversal potential of I_K1 _to -85 mV [data not shown] and the mean current of I_K1 _was -1.27 ± 0.67 pA/pF. Hence, there appears to be a large Na^+^-dependent inward current in chick embryonic cardiac cells accounting for apparently small reversal potential seen in Figs. 5 and 6.

### Anandamide has no effect on I_k1 _current in ED11 ventricular myocytes

To examine whether 3 μM anandamide modulates other K^+ ^channels, we compared the effects of barium and anadamide on current-voltage relationships of ED11 ventricular myocytes where both inwardly rectifying and delayed rectifier K^+ ^currents are present.

Current recordings and I-V relationships in ED11 ventricular myocytes showed the activation of inwardly rectifying K^+ ^current (I_K1_) at negative potentials and the activation of delayed rectifier I_K _at positive potentials (Figs. 6A and 6B). Anandamide (3 μM) had no effect on I-V relationships, indicating that anandamide had no effect on either I_K1 _or I_K _(Fig. 6B and 6C). On the other hand, adding 100 μM Ba^2+ ^significantly inhibited I_K1 _while leaving I_K _unaffected (Fig. 6B). The currents at -80 mV in the absence and presence of anandamide were -4.5 ± 1.1 and -4.3 ± 0.9 pA/pF, respectively, and were not significantly different, while barium inhibited a large current at this potential in the same cells. Anandamide-sensitive K^+ ^current at -80 mV was 0.07 ± 0.48 pA/pF, while Ba^2+^-sensitive current was 1.95 ± 0.86 pA/pF (Fig. 6C, n = 11). It is apparent from these results that anandamide has no effect on inward rectifier, I_K1_.

## Discussion

In the present study, we demonstrate that the functional expression of an acid-sensitive 2P domain K^+ ^channel, TASK-1, is developmentally regulated in chick embryonic heart. TASK-1 is initially expressed throughout the myocardium in the early heart tube. Expression of TASK-1 remained in the atria, but was down-regulated later in the ventricles. TASK-3 is consistently present at all chick heart developmental ages. Consistent with expression studies, TASK-1 currents are present in tubular heart, but the current densities are the highest later in the developing atria. In contrast, I_K1 _has 4-5 times lesser current density in ED11 atrial myocytes than in ED11 ventricular myocytes that have only small TASK-1 currents. Our electrophysiological and TASK-1 mRNA expression data clearly demonstrate the presence of TASK-1 channels in native embryonic cardiac myocytes and its contribution to background K^+ ^conductance. The developmental regulation of TASK-1 expression suggests that TASK-1 is important both in heart development and in regulating membrane potentials.

### Developmental expression and regulation of TASK-1 channel in chick embryonic heart

The distribution of TASK-1 during heart development may imply its functional role. Compared to TASK-3, TASK-1 is ubiquitously expressed earlier in heart development. TASK-1 expression is down-regulated later in development in ventricle but is maintained in later developmental stages in atria. On the other hand, TASK-3 channels are more uniformly expressed in all chick heart developmental stages.

The distinguishing characteristics of TASK channels (mainly TASK-1 and TASK-3) are their sensitivity to external pH. Here we showed an acid-sensitive background K^+ ^current in the developing chicken heart having similar characteristics that are consistent with TASK-1 channels (Fig. 3A). However, in the symmetrical K^+ ^and chloride-free solutions, I-V relations of TASK currents show strong inward rectification, closely resembling the I-V relationships of TASK-1 single channel conductance and TASK-1/TASK-3 channels [16]. TASK currents also become bigger at negative potentials. These data suggest the possibility that TASK-1 current is masked by a Cl^- ^current when measured in symmetrical KCl solutions.

The difference in pH sensitivity of TASK-1 and TASK-3 (pKa of 7.3 and 6.7 respectively) has been successfully used to distinguish these channels [16]. In addition, anandamide and Zn^2+ ^have been used as relatively selective inhibitors of cloned TASK-1 and TASK-3 currents [17,18]. Although both anandamide and zinc are endogenous ion channel modulators and at higher concentrations they could inhibit other K2P channels, the concentrations used here are to selectively inhibit TASK-1 or TASK-3 channels. Therefore, TASK currents in chick embryonic heart that show the sensitivity to both anadamide and external protons are mostly due to TASK-1 channels.

Anandamide-sensitive TASK-1 currents comprise only about two-thirds of the acid-sensitive background K^+ ^currents in the ED11 atrium. One possibility is because the inhibition of TASK-1 by anandamide is less effective at negative potentials (Fig. 4). Still, we could not preemptively exclude the contribution of other acid sensitive K2P channels. Among the five members of TASK family, TASK-5 is a non-functional channel [21] and TASK-4 actually belongs to the TALK subfamily and is activated at alkaline pH (>pH 8.0). TASK-2 is strongly expressed in epithelial tissues, particular in the kidney to regulate cell volume but, is only weakly expressed in the adult heart [22]. Therefore, the contribution from other acid sensitive K2P channels should be relatively small.

It is surprising that although TASK-3 mRNA was present at a relatively higher level and was expressed equally at all developmental stages (Fig. 1B) its contribution to TASK currents is quite small under our experimental conditions (Fig. 3B). 100 μM Zn^2+ ^does not induce a significant inhibition of TASK currents in chick ED11 atrial myocytes (Fig. 4C). One possible explanation is that TASK-3 might form functional heterodimers with TASK-1 in native embryonic cardiac myocytes because heterologous TASK-1/TASK-3 channel has an intermediate pH sensitivity and low sensitivity to ruthenium red and Zn^2+ ^(16-17). In fact, in TASK-1 knockout mice, TASK currents, mostly contributed by homodimers of TASK-3 channels, are greatly inhibited by 100 μM Zn^2+ ^in cerebellar granule neurons [23]. Functional TASK-1/TASK-3 heterodimers have been reported in cerebellar granule cells [16,24].

### Possible functional role of TASK-1 in embryonic cardiac excitability

In the early tubular heart, all myocytes exhibit spontaneous electrical activity similar to adult sinoatrial node cells and have a relatively depolarized maximum diastolic potential. The spontaneous activity or automaticity of the early tubular heart is a consequence of the less negative maximum diastolic potential that is due to the lack of I_K1_, higher P_Na_/P_K _permeability, and higher membrane resistance of embryonic myocytes which are similar to SA nodal cells in adult hearts [11]. With a higher membrane resistance, a small current such as TASK-1 current could play a significant role in modulating embryonic cardiac action potentials.

In chick heart development, a significant hyperpolarizing change in RMP in ventricular myocytes occurs between ED5 and ED7 [25]. In the developing chick atria, RMP does not change significantly between ED9 and ED19, and atria have a more depolarized RMP (-63 mV) compared to the ventricle (-80 mV) [26]. Data shown here indicates that TASK-1 expression is down-regulated during the period when the RMP becomes more hyperpolarized and I_K1 _is evidently present in developing chick ventricle. This indicates that I_K1 _is a dominant contributor to RMP in ventricle after about ED5. In embryonic atria where there is a lack of I_K1_, temperature-sensitive TREK channels have been shown to play a very important role in setting RMP [27]. Thus, TASK-1 channels are unlikely to play a dominant role in setting RMP although they are maintained in ED11 atrial myocytes. However, unlike TREK channels, TASK-1 channels do not have strong temperature sensitivity [28]. During avian heart development, the temperature environment may vary significantly. Therefore when temperature dependent inactivation of TREK channels occurs and there is a lack of the normally dominant background K^+ ^current, I_K1_, TASK-1 channels remain active and could play a role in maintaining the stability of membrane potentials.

In general, TASK-1 may contribute to setting the maximum diastolic potential, to the repolarization of action potentials, and to shortening the duration of action potential as has been shown for TASK-1 activity in adult rat and mouse ventricular myocytes [8,29].

## Conclusion

In this study, we were able to demonstrate the functional expression of TASK-1 in the early tubular heart and in the embryonic atrial myocytes. The expression of TASK-1 is developmentally regulated in chick embryonic heart. Functional expression of TASK-1 is likely to be a contributor to background membrane conductance in cardiac myocytes from early tubular heart to embryonic atrial myocytes.

## List of abbreviations

TASK: TWIK-related Acid Sensitive Potassium Channels; RMP: resting membrane potential; AP: Action Potential; ED: Embryonic Day; K2P: Two-pore Domain Potassium Channels; NTB: Nitro-Blue Tetrazolium Chloride; BCIP: 5-Bromo-4-Chloro-3'-Indolyphosphate p-Toluidine Salt; TEA: Tetraethylammonium; 4-AP: 4-Aminopyridine; EGTA: Ethylene Glycol-bis [β-aminoethyl ether] N,N,N'N'-tetraacetric acid; DMSO: dimethylsulfoxide.

## Competing interests

The authors declare that they have no competing interests.

## Authors' contributions

HZ designed, performed experiments and prepared a draft of the manuscript. JP performed some of patch clamp experiments. NS and and TLC revised the drafted manuscript.
